# Supplementary material for: Elevated Photovoltaic Performance in Medium Bandgap Copolymers Composed of Indacenodi-thieno[3,2-b]thiophene and Benzothiadiazole Subunits by Modulating the π-Bridge
Source: Polymers (Basel). 2020 Feb 7;12(2):368. doi: 10.3390/polym12020368 (PMC7077401; doi:10.3390/polym12020368)
Supplement: Supplementary file 1 [file polymers-12-00368-s001.pdf]

Supplementary Material

Elevated Photovoltaic Performance in Medium Bandgap Copolymers Composed of Indacenodithieno[3,2-*b*]thiophene and Benzothiadiazole Subunits by Modulating the *π*-bridge

Lili An, Junfeng Tong, Yubo Huang, Zezhou Liang, Jianfeng Li, Chunyan Yang, Xunchang Wang

Synthesis of $DTBTBr_2$, $TBTBr_2$ and $TFBTBr_2$ [S1,S2]

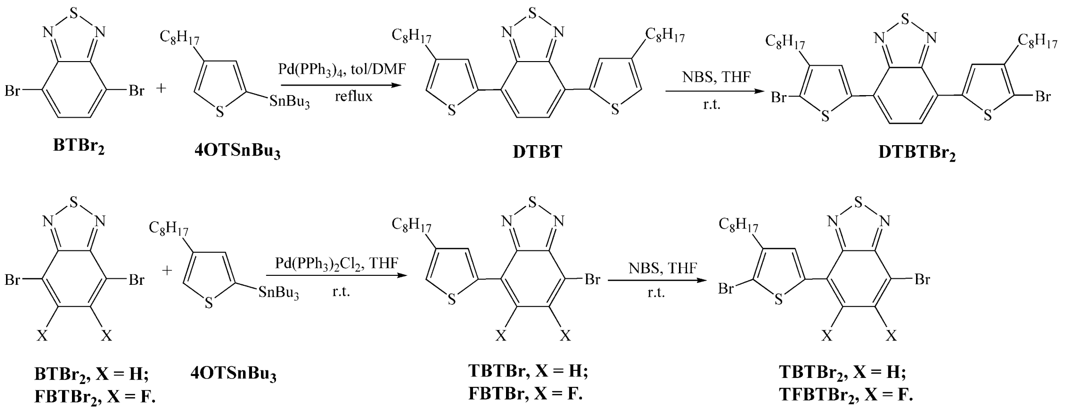

Scheme S1 Synthesis of dibromide $DTBTBr_2$, $TBTBr_2$ and $TFBTBr_2$.

4,7-Bis(4-octylthien-2-yl)benzo[*c*][1,2,5]thiadiazole (DTBT)

In a 250 mL two-neck flask, $Pd(PPh_3)_4$ (593 mg, 0.513 mmol) was added to a stirring 84 mL toluene and 17 mL *N,N*-dimethylformamide (DMF) mixture solution of $BTBr_2$ (5.00 g, 17.0 mmol) and 4-(2-octyl)-2-tributyltinthiophene (24.0 g, 44.5 mmol). The mixture was stirred at 105 ^{o}C for 18 h under Ar atmosphere. Then the reaction mixture was poured into the cold PE, the solid was produced. And the solid was obtained by vacuum filtration, the residue was re-dissolved in chloroform, and extracted by chloroform (CF) and washed with water and brine. After drying by anhydrous Na_2SO_4, the solvent was removed at reduced pressure. The crude product was purified by chromatography (silica gel, 200–300 mesh) using PE:$CHCl_3$ (10:1, V/V) as the eluent and further recrystallized from EtOH to afford 8.70 g red solid. Yield: 86%. M.p.: 53~54 ^{o}C. ^{1}H NMR ($CDCl_3$, 400 MHz), δ (ppm): 7.98 (s, 2H), 7.83 (s, 2H), 7.04 (s, 2H), 2.69 (d, ^3J = 7.2 Hz, 4H), 1.72−1.29 (m, 24H), 0.88 (t, ^3J = 7.2 Hz, 6H). Anal. Calcd for $C_{30}H_{40}N_2S_3$: C, 68.65%; H, 7.68%; N, 5.34%. Found, C, 68.45%; H, 7.56%; N, 5.54%.

4,7-Bis(5-bromo-4-octylthien-2-yl)benzo[*c*][1,2,5]thiadiazole ($DTBTBr_2$)

Into a 100 mL THF solution of DTBT (1.50 g, 2.70 mmol), *N*-bromosuccinimide (NBS, 1.10 g, 6.00 mmol) dissolved in THF (10 mL) was added slowly under an ice-water bath, and the reaction mixture was stirred at room temperature for 6 h under dark. The react mixture was poured into 100 mL water and extracted with $CHCl_3$, then washed with brine and water 3 times, respectively. The combined organic layers were dried over anhydrous Na_2SO_4, and the solvents were removed by rotary evaporation. Finally, the crude compound was purified by column chromatography (silica gel 200–300 mesh; eluent: PE:$CHCl_3$ = 20:1) and then recrystallized from EtOH to yield 1.60 g deep-red solid. Yield: 84%. M.p.: 72~74 ^oC. ^1H NMR ($CDCl_3$, 400 MHz), δ (ppm): 7.77 (s, 2H), 7.74 (s, 2H), 2.64 (t, ^3J = 7.2 Hz, 4H), 1.67 (m, 4H), 1.45−1.20 (m, 20H), 0.88 (t, ^3J = 7.2 Hz, 6H). Anal. Calcd for $C_{30}H_{38}Br_2N_2S_3$: C, 52.78%; H, 5.61%; N, 4.10%. Found, C, 52.79%; H, 5.64%; N, 4.09%.

4-Bromo-7-(4-octylthien-2-yl)-2,1,3-benzothiadiazole (TBTBr).

In a 250 mL three-neck flask, $Pd(PPh_3)_2Cl_2$ (840 mg, 1.20 mmol) was added to a stirring 150 mL THF solution of 4,7-dibromo-2,1,3-benzothiadiazole (17.00 g, 57.8 mmol) and 4-octyl-2-(tributylstannyl)thiophene (30.9 g, 63.6 mmol) at room temperature. After stirring for 24 h, the solvent was removed under reduced pressure, then 200 mL cold petroleum ether (PE) was added into the residue and filtrated under reduced pressure. The obtained solid was washed with cold PE three times and purified by chromatography using PE/dichloromthane (5:1, V/V Silica gel, 300–400 mesh), affording 15.9 g yellow solid with a yield of 67.3%. M.p.: 51–52 ℃. ^1H NMR (400 MHz, $CDCl_3$) 7.95 (s, 1H), 7.82 (d, ^3J = 8.0 Hz, 1H), 7.67 (d, ^3J = 8.0 Hz, 1H), 7.06 (s, 1H), 2.68 (t, ^3J = 7.6 Hz, 2H), 1.69 (m, 2H), 1.40–1.25 (m, 10H), 0.88 (t, ^3J = 6.8 Hz, 3H). Alal. Calcd for $C_{18}H_{21}BrN_2S_2$: C, 52.81, H, 5.17, N, 6.84%; Found: C, 52.69%; H, 5.02%; N, 6.99%.

4-Bromo-7-(5-bromo-4-octylthien-2-yl)benzo[*c*][1,2,5]thiadiazole ($TBTBr_2$)

Into a 50 mL THF solution of TBTBr (5.00 g, 12.2 mmol), *N*-bromosuccinimide (NBS, 2.60 g, 14.6 mmol) was added slowly and stirred at r.t. for 6 h under dark. The reaction was monitored by TLC. Then, the react mixture was poured into 100 mL water and extracted with $CHCl_3$, then washed with brine and water 3 times, respectively. The combined organic layers were dried over anhydrous Na_2SO_4, and the solvents were removed by rotary evaporation. Finally, the crude compound was purified by column chromatography (silica gel: 200–300 mesh; eluent: PE:DCM = 8:1) and isolated as a yellow solid of 15.90 g. Yield 67.3%. M.p.:

73–74 °C. ^{1}H NMR ($CDCl_3$, 400 MHz), δ (ppm): 7.83 (d, ^3J = 7.6 Hz, 1H), 7.75 (s, 1H), 7.62 (d, ^3J = 7.6 Hz, 1H), 2.63 (t, ^3J = 7.6 Hz, 2H), 1.67 (m, 2H), 1.40−1.25 (m, 10H), 0.88 (t, ^3J = 6.4 Hz, 3H). Anal. Calcd for $C_{18}H_{20}Br_2N_2S_2$: C, 44.27%; H, 4.13%; N, 5.74%. Found, C, 44.24%; H, 4.01%; N, 5.84%.

4-Bromo-7-(4-octylthien-2-yl)-5,6-difluorobenzo[*c*][1,2,5]thiadiazole (TFBTBr)

The coupling procedure of TFBTBr was similar to the synthesis of TBTBr, except that reagents was replaced by $FBTBr_2$ (3.03 g, 9.18 mmol) and 4-octyl-2-tributyltinthiophene (4.85 g, 10.0 mmol). The target compound was obtained as yellow solid of 2.87 g. Yield 71%. M.p.: 70–72 °C. ^{1}H NMR ($CDCl_3$, 400 MHz), δ (ppm): 8.12 (s, 1H), 7.20 (s, 1H), 2.71 (t, ^3J = 7.2 Hz, 2H), 1.70 (m, 2H), 1.40−1.25 (m, 10H), 0.88 (t, ^3J = 6.4 Hz, 3H). Anal. Calcd for $C_{18}H_{19}BrF_2N_2S_2$: C, 48.54%; H, 4.30%; N, 6.29%. Found, C, 48.44%; H, 4.21%; N, 6.64%.

4-Bromo-7-(5-bromo-4-octylthien-2-yl)-5,6-difluorobenzo[*c*][1,2,5]thiadiazole ($TFBTBr_2$)

The bromination procedure of $TFBTBr_2$ was similar to the synthesis of $TBTBr_2$, except that reagents were replaced by TFBTBr (2.50 g, 5.61 mmol) and NBS (1.20 g, 6.73 mmol). The target compound was obtained as yellow solid of 2.21 g. Yield 75%. M.p.: 89–90 °C. ^{1}H NMR ($CDCl_3$, 400 MHz), δ (ppm): 7.94 (s, 1H), 2.66 (t, ^3J = 7.6 Hz, 2H), 1.66 (br, 2H), 1.40−1.25 (m, 10H), 0.86 (t, ^3J = 6.4 Hz, 3H). Anal. Calcd for $C_{18}H_{18}Br_2F_2N_2S_2$: C, 41.24%; H, 3.46%; N, 5.34%. Found, C, 41.04%; H, 3.21%; N, 5.52%.

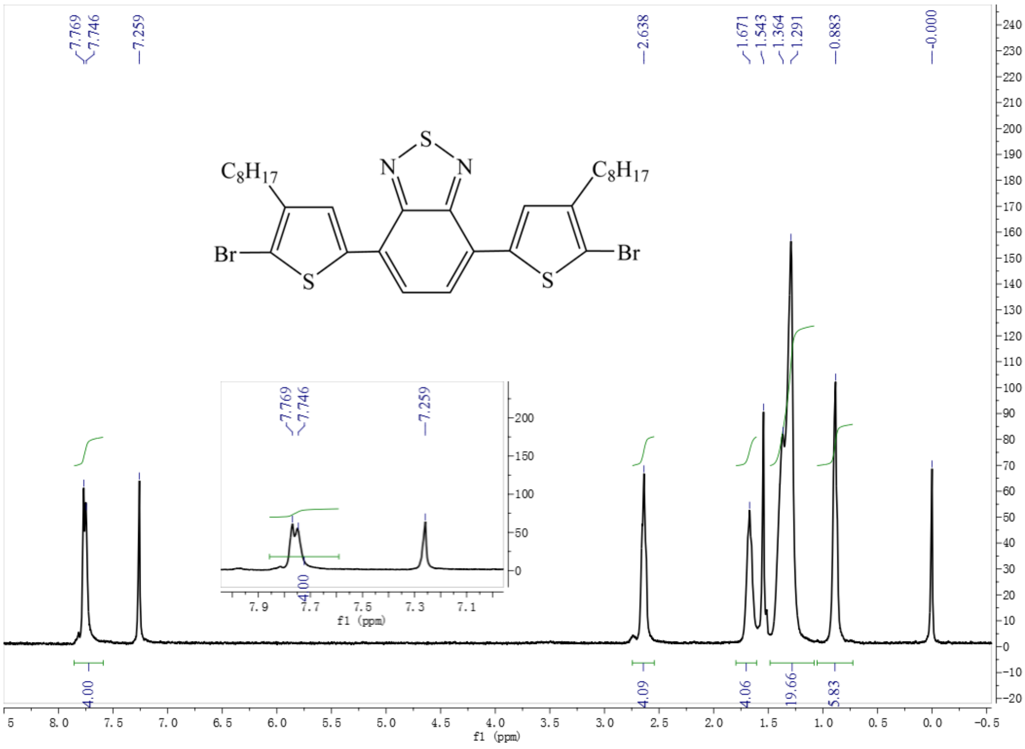

Fig. S1. ^{1}H NMR spectrum of $DTBTBr_2$ in $CDCl_3$

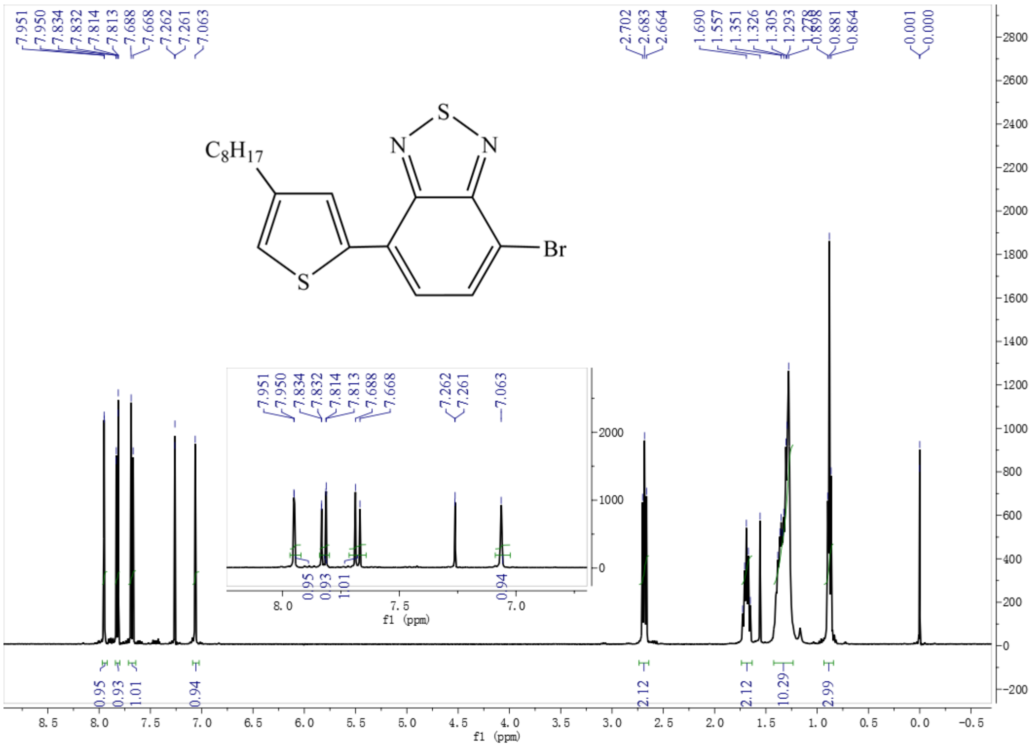

Fig. S2. ^{1}H NMR spectrum of TBTBr in $CDCl_3$

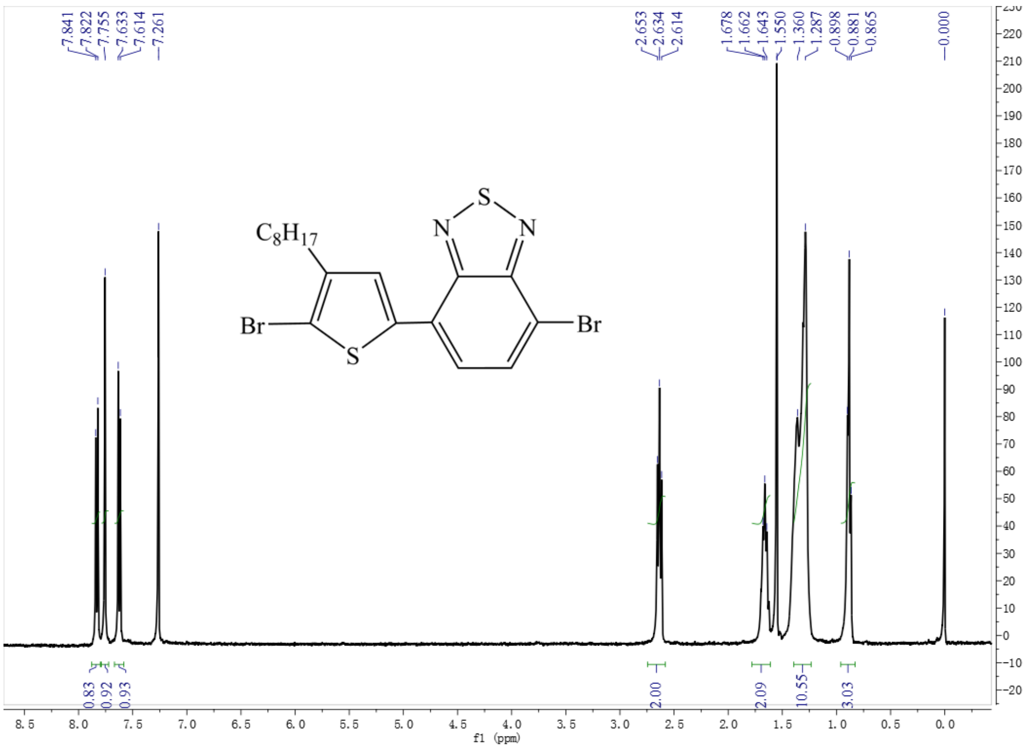

Fig. S3. ^{1}H NMR spectrum of $TBTBr_2$ in $CDCl_3$

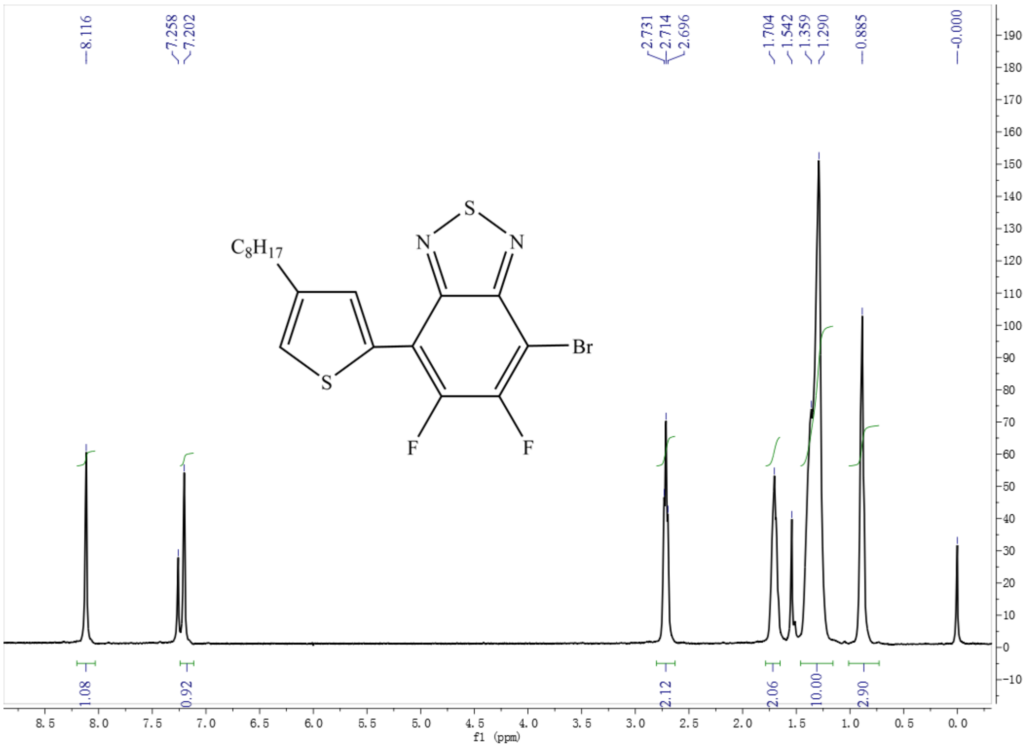

Fig. S4. ^{1}H NMR spectrum of TFBTBr in $CDCl_3$

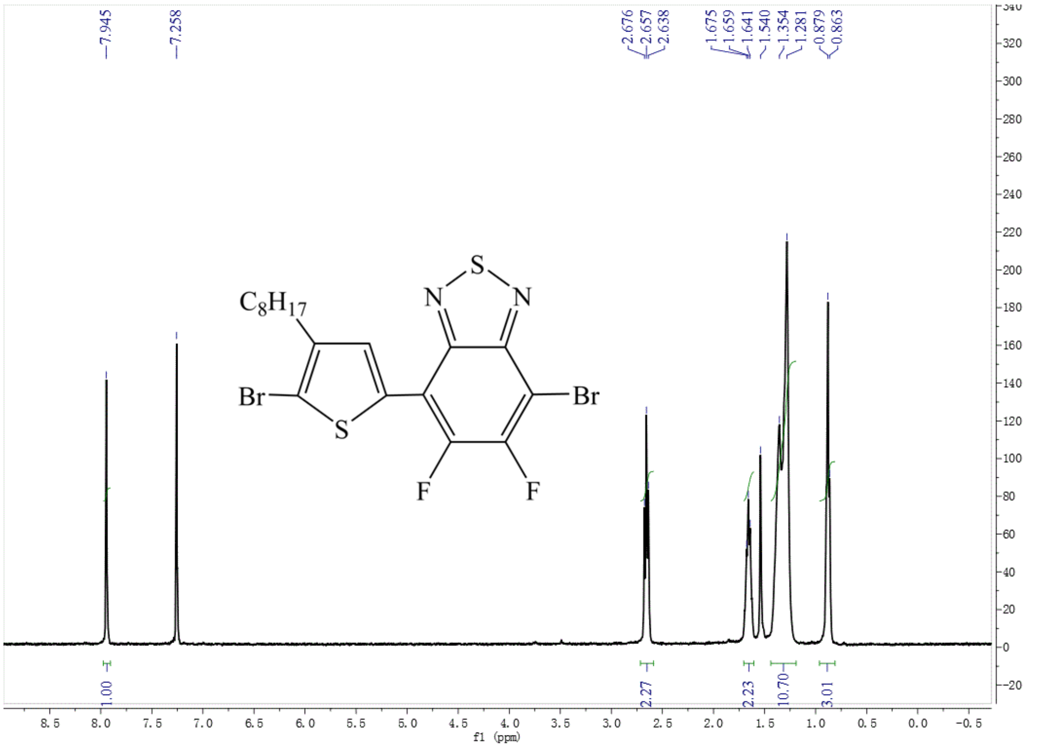

Fig. S5. ^{1}H NMR spectrum of $TFBTBr_2$ in $CDCl_3$

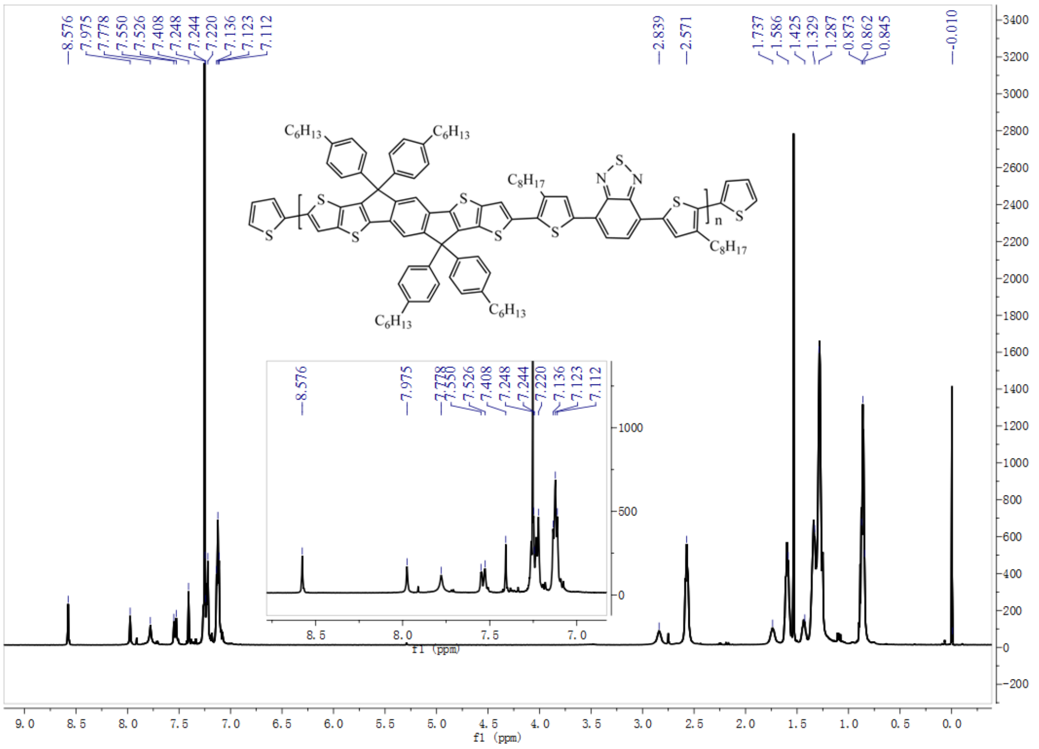

Fig. S6 ^{1}H NMR spectrum of PIDTT-DTBT in $CDCl_3$

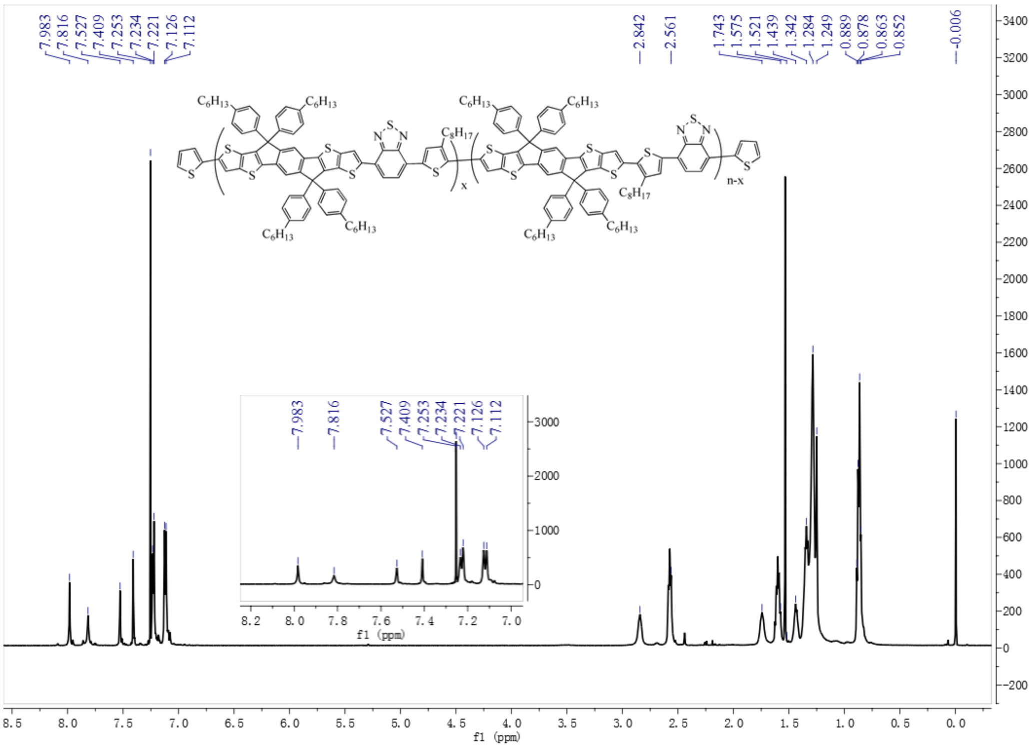

Fig. S7 ^{1}H NMR spectrum of PIDTT-TBT in $CDCl_3$

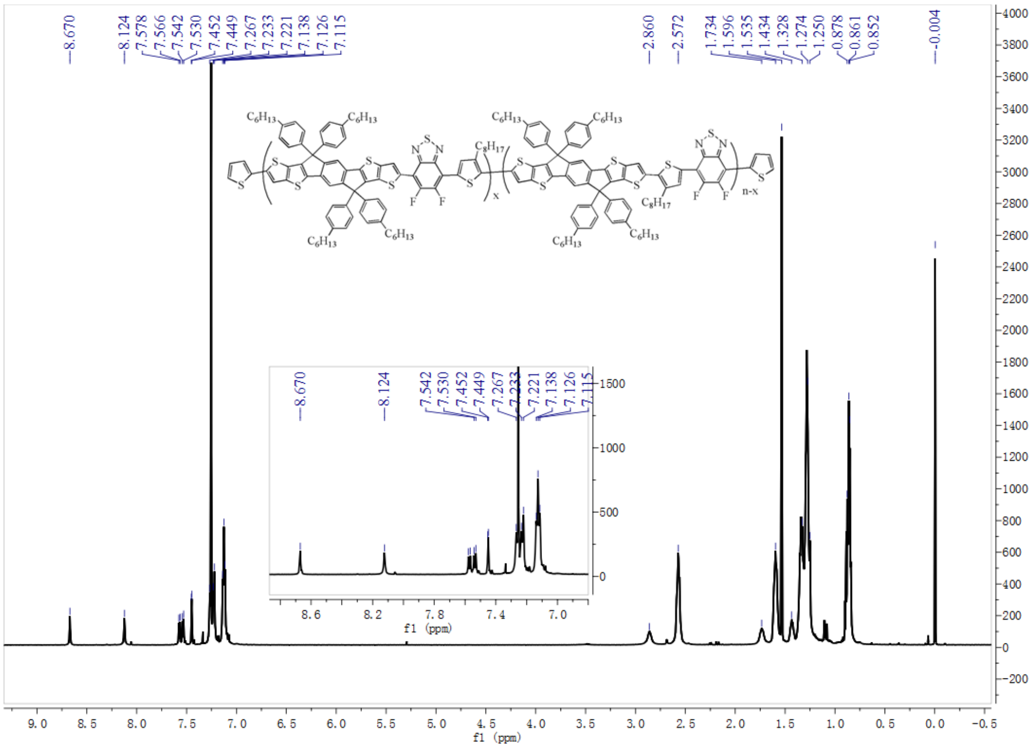

Fig. S8 ^{1}H NMR spectrum of PIDTT-TFBT in $CDCl_3$.

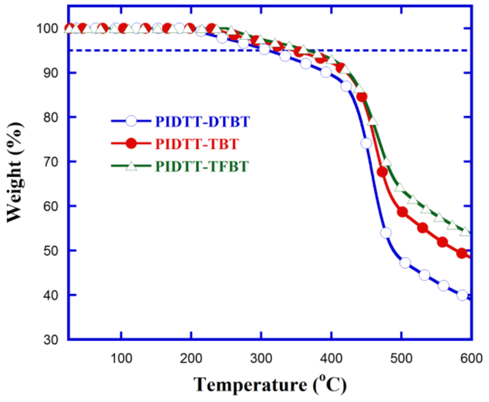

Fig. S9 TGA curves of PIDTT-DTBT, PIDTT-TBT and PIDTT-TFBT.

Table S1. Yields, GPC data, thermal properties for the studied copolymers.

Polymer	Yield (%)	M_n (kDa)	M_w (kDa)	PDI	T_d (℃）
**PIDTT-DTBT**	98	32.4	51.2	1.8	310
**PIDTT-TBT**	93	31.2	59.3	1.9	346
**PIDTT-TFBT**	92	34.4	72.2	2.1	366

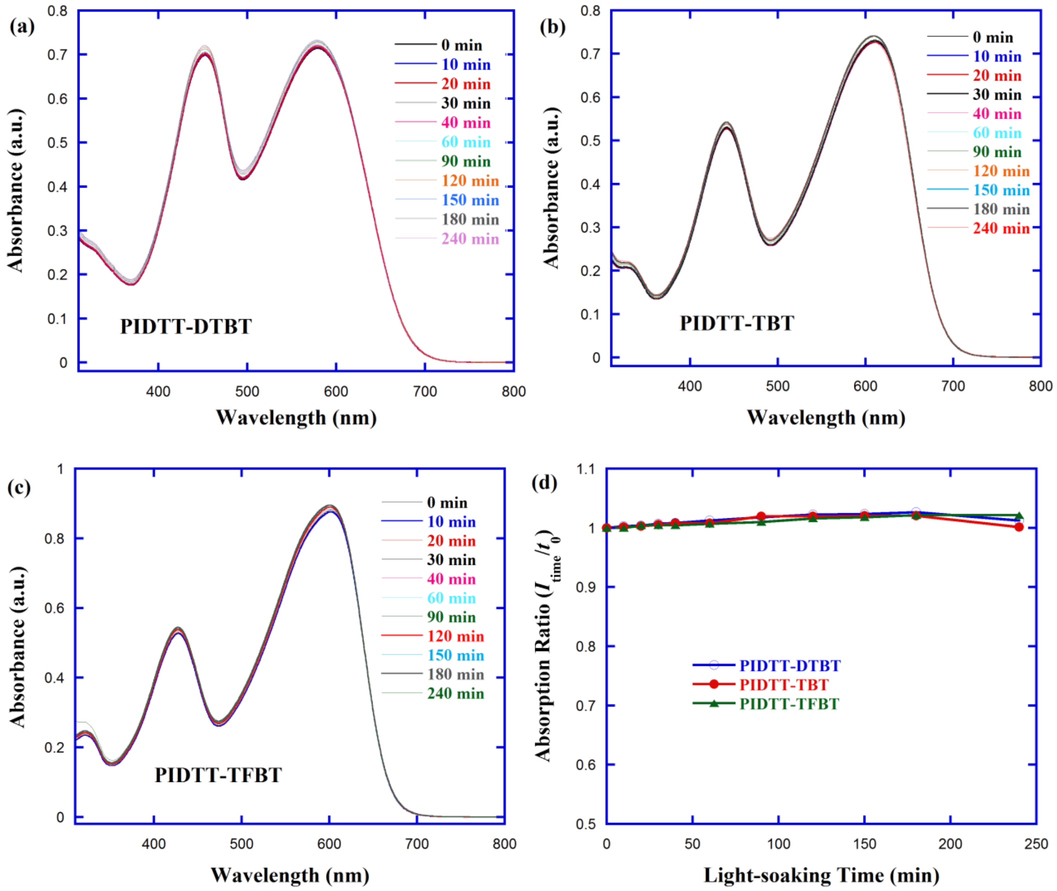

Fig. S10 UV-vis absorption spectra changes after illumination of PIDTT-DTBT (a), PIDTT-TBT (b) and PIDTT-TFBT (c) in CB solution and a summary of light absorption intensity changes at the peak of each polymer as a function of light-soaking time (d).

Table S2. The photovoltaic performance of the PSCs devices under varied fabrication processes.

Active layer (w:w)	DIO	V_{OC} (V)	J_{SC} (mA cm^{-2})	*FF* (%)	PCE(%)	R_{SH} (Ω m^2)[a]	R_S (Ω m^2)[a]
**PIDTT-DTBT**/$PC_{61}BM$ (1:1)	0%	0.80	3.60	30.16	0.87	305	97.0
**PIDTT-DTBT**/$PC_{61}BM$ (1:2)	0%	0.81	5.51	45.67	2.05	565	43.4
**PIDTT-DTBT**/$PC_{61}BM$ (1:3)	0%	0.83	6.41	36.88	1.96	267	44.2
**PIDTT-DTBT**/$PC_{61}BM$ (1:2)	3%	0.84	5.57	37.37	1.75	293	44.2
**PIDTT-DTBT**/$PC_{71}BM$ (1:2)	0%	0.82	5.76	41.15	1.94	429	28.5
**PIDTT-TBT**/$PC_{61}BM$ (1:1)	0%	0.88	3.48	32.50	1.00	360	71.5
**PIDTT-TBT**/$PC_{61}BM$ (1:2)	0%	0.87	10.44	49.30	4.48	434	13.4
**PIDTT-TBT**/$PC_{61}BM$ (1:3)	0%	0.86	5.19	41.90	1.88	395	26.4
**PIDTT-TBT**/$PC_{61}BM$ (1:2)	3%	0.88	10.54	53.14	4.94	386	11.8
**PIDTT-TBT**/$PC_{71}BM$ (1:2)	3%	0.88	11.08	59.60	5.84	604	7.70
**PIDTT-TFBT**/$PC_{61}BM$ (1:1)	0%	0.90	3.81	36.85	1.25	425	31.5
**PIDTT-TFBT**/$PC_{61}BM$ (1:2)	0%	0.94	5.63	49.08	2.60	474	19.7
**PIDTT-TFBT**/$PC_{61}BM$ (1:3)	0%	0.94	5.44	47.58	2.44	485	20.5
**PIDTT-TFBT**/$PC_{61}BM$ (1:2)	3%	0.95	5.95	51.54	2.92	536	14.9
**PIDTT-TFBT**/$PC_{71}BM$ (1:2)	3%	0.95	5.44	51.86	2.67	557	14.8

[a]The values in the parentheses are the integrated currents obtained from the EQE curves.

[b]Shunt resistance (R_{SH}) and series resistance (R_S) are deduced from the inverse slope at $V = 0$ and $V = V_{OC}$ in the *J*−*V* curves under illumination.

Table S3 Hole mobilities of the optimized devices measured by SCLC model.

Active layer	Ratios/Additive	Thickness (nm)	Slope	μ_h (cm^2 V^{-1} s^{-1})
PIDTT-DTBT:$PC_{61}BM$	1:2/0%DIO	98	23.74	1.78×10^{-4}
PIDTT-TBT:$PC_{71}BM$	1:2/3%DIO	100	47.85	7.67×10^{-4}
PIDTT-TFBT:$PC_{61}BM$	1:2/3%DIO	105	36.95	5.29×10^{-4}

Table S4 Electron mobilities of the optimized device measured by SCLC model.

Active layer	Ratios/Additive	Thickness (nm)	Slope	μ_e (cm^2 V^{-1} s^{-1})
PIDTT-DTBT:$PC_{61}BM$	1:2/0%DIO	115	0.41	8.56×10^{-8}
PIDTT-TBT:$PC_{71}BM$	1:2/3%DIO	118	0.86	4.07×10^{-7}
PIDTT-TFBT:$PC_{61}BM$	1:2/3%DIO	100	2.30	1.77×10^{-6}

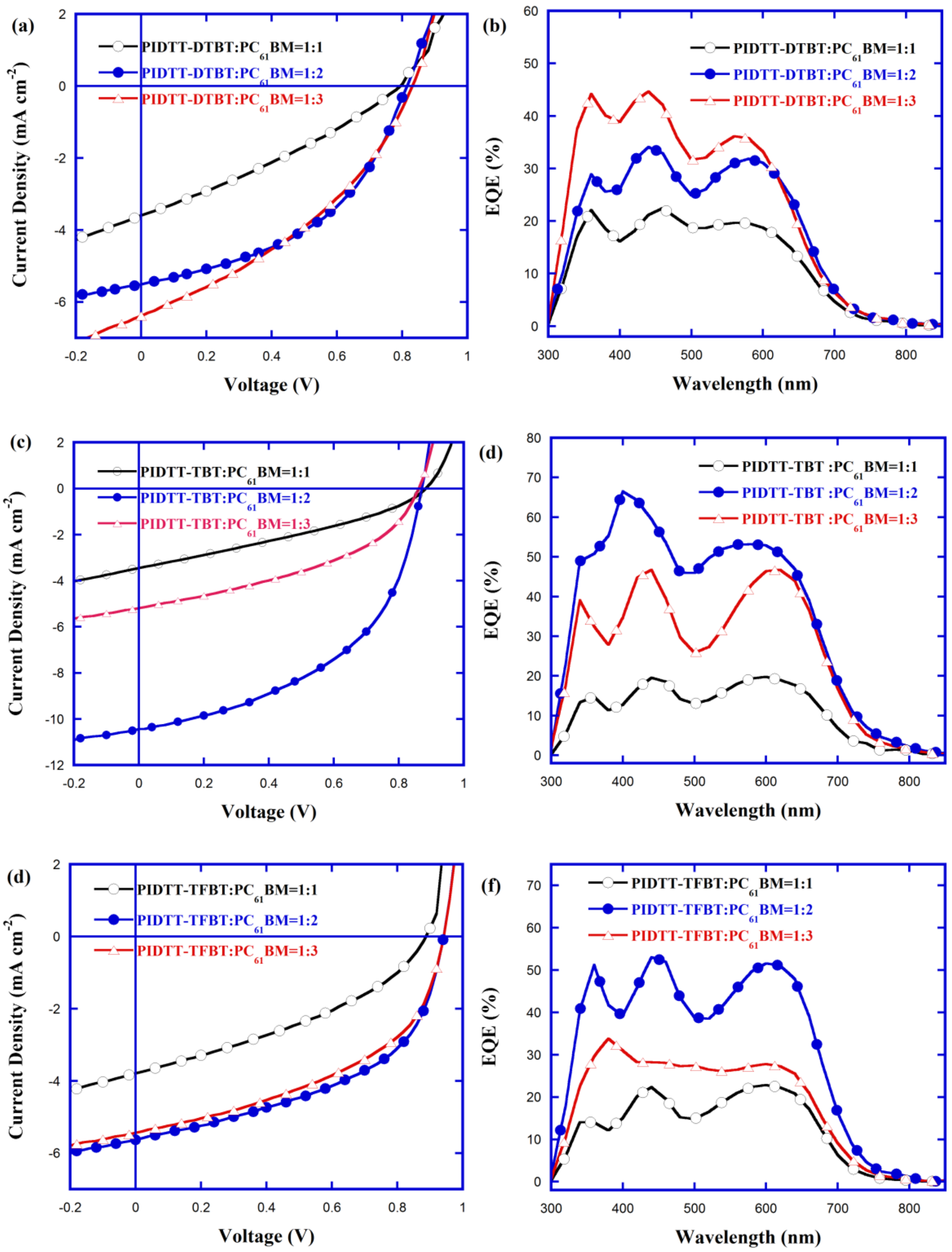

Fig. S11 The *J*-*V* curves of the studied copolymers with different weight ratio to $PC_{61}BM$ and EQE spectra of corresponding PSCs.

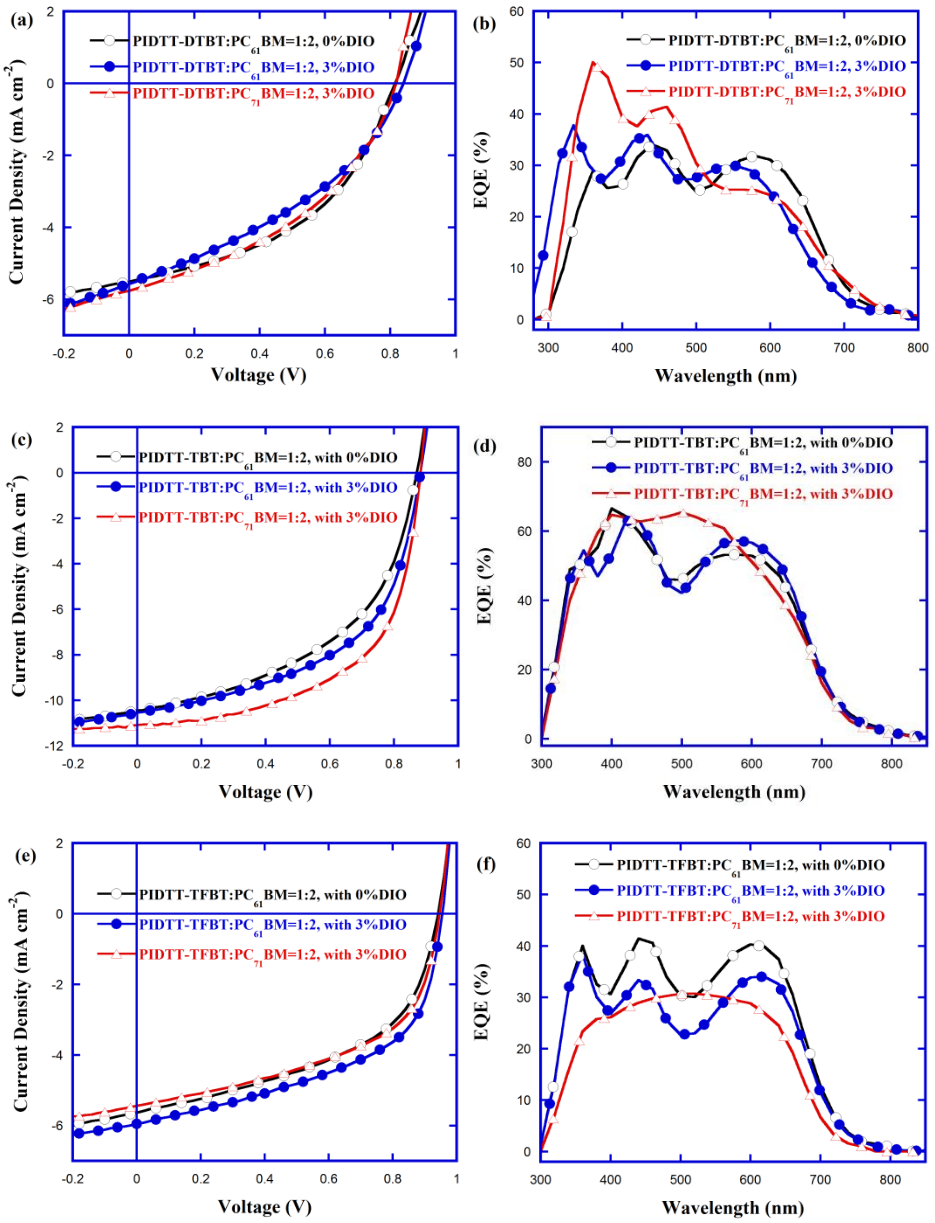

Fig. S12 The *J*-*V* curves of polymers without and with 3%DIO, $PC_{61}BM$ replacing with $PC_{71}BM$ and EQE spectra of corresponding PSCs.

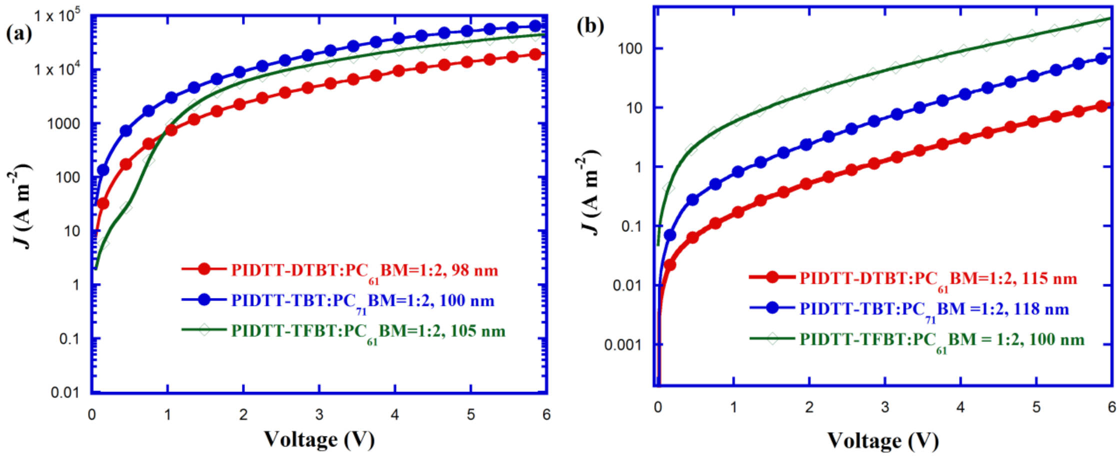

Fig. S13. *J-V* curves of hole-only (a) and electron-only (b) devices for the studied copolymers.

Reference

[S1] Li, J.; Tong, J., Zhang, P., Yang, C., Chen, D., Zhu, Y., Xia, Y.; Fan, D. Synthesis and photovoltaic properties of alternating conjugated polymers derived from thiophene-benzothiadiazole block and fluorene/indenofluorene units. *Bull. Korean Chem. Soc*. **2014**, 35, 505–512.

[S2] Zhang, X.; Wang, F.; Tong, J.; Zhang, M.; Guo, P.; Li, J.; Xia, Y.; Wang, C.; Wu, H. Systematically investigating the influence of inserting alkylthiophene spacers on the aggregation, photo-stability and optoelectronic properties of copolymers from dithieno[2,3-*d*:2′,3′-*d*′]benzo[1,2-*b*:4,5-*b*′]di-thiophene and benzothiadiazole derivatives. *Polym. Chem.* **2019**, *10*, 972–982.
